# Functional criticality in the human brain: Physiological, behavioral and neurodevelopmental correlates

**DOI:** 10.1371/journal.pone.0213690

**Published:** 2019-03-08

**Authors:** Lili Jiang, Kaini Qiao, Danyang Sui, Zhe Zhang, Hao-Ming Dong

**Affiliations:** 1 CAS Key Laboratory of Behavioral Science, Institute of Psychology, Beijing, China; 2 Lifespan Connectomics and Behavior Team, Institute of Psychology, Chinese Academy of Sciences, Beijing, China; 3 Department of Psychology, University of Chinese Academy of Sciences, Shijingshan, Beijing, China; Hong Kong Baptist University, HONG KONG

## Abstract

Understanding the critical features of the human brain at multiple time scales is vital for both normal development and disease research. A recently proposed method, the vertex-wise Index of Functional Criticality (vIFC) based on fMRI, has been testified as a sensitive neuroimaging marker to characterize critical transitions of human brain dynamics during Alzheimer’s disease progression. However, it remains unclear whether vIFC in healthy brains is associated with neuropsychological and neurophysiological measurements. Using the Nathan Kline Institute/Rockland lifespan cross-sectional datasets and openfMRI single participant longitudinal datasets, we found consistent spatial patterns of vIFC across the entire cortical mantle: the inferior parietal and the precuneus exhibited high vIFC. On a time scale of years, we observed that vIFC increased with age in the left ventral posterior cingulate gyrus. On a time scale of days and weeks, vIFC demonstrated the capacity to identify a link between anxiety and pulse. These results showed that vIFC can serve as a useful neuroimaging marker for detecting physiological, behavioral, and neurodevelopmental transitions. Based on the criticality theory in nonlinear dynamics, the current vIFC study sheds new light on human brain studies from a nonlinear perspective and opens potential new avenues for normal and abnormal human brain studies.

## Introduction

The human brain is one of the most complicated dynamical systems. It varies at multiple temporal and spatial scales, from milliseconds and microns in neuron firing to seconds and centimeters in functional magnetic resonance imaging blood oxygen level-dependent time series of brain regions and even to days, weeks, months and years during lifespan development. Criticality is a state of being scale-free and may accommodate this multiscale phenomenon in the human brain. Additionally, there have been studies showing that the human brain works near criticality to accomplish the transitions of task states [1–4]. Is there any critical transition or abrupt change in human brain dynamics at some spatiotemporal scale that may indicate neurodevelopment, physiology or behavior? vIFC, a recently proposed vertex-wise Index of Functional Criticality of human brain resting state fMRI time series [5], provides us with a possible opportunity to examine the critical transitions of the human brain and their associations with neurodevelopment, physiology, and behavior. vIFC has been proposed based on nonlinear dynamical theory as an efficient neuroimaging marker that indicates probabilities that a critical transition occurs in the absence of knowledge of the details of the realistic network connections [6]. In more detail, vIFC was designed to integrate three properties of a center manifold (subnetwork or a group of variables) in the abstract phase space of a complicated human brain network: increased within-group correlations, increased temporal variations and decreased between-group correlations. The dynamical network biomarker method deriving from the three properties has been used for predicting critical transitions of diseases based on biochemical and genomic data, including respiratory disease [7], depression [8], episodic migraine [9] and type 1 diabetes [10].

In fact, critical transitions have been suggested to exist widely in ecosystems [11], climate systems [12], economics and global finance [13]. Although the human brain is a rather complicated system and varies at multiple time scales, few studies have focused on critical transitions in the human brain. Applications of resting-state fMRI have greatly promoted the elucidation of the developmental mechanisms of human brain structure and function from a time scale of years. Converging neuroimaging evidence suggests that a local-to-distributed evolution of organization occurs during development [14–17]. This conclusion, however, was deduced from a linear hypothesis and statistics of phenomenal descriptions based on fMRI scanning data of the human brain. Although indexes such as regional homogeneity (ReHo) [18], degree centrality, and eigenvector centrality [19,20] have been widely applied in neuroimaging studies, they were heuristically raised without a theoretical basis and are difficult to understand in terms of their biological and theoretical implications. In contrast, vIFC analysis has a solid theoretical background and is designed to represent the probabilities that critical transitions occur in the human brain during a certain time period. Compared with traditional biomarkers evaluating the system state in a rather static and linear manner, vIFC was used to predict incoming critical transitions from the nonlinear hypothesis of the human brain.

Could vIFC reflect physiological, behavioral or neurodevelopmental properties at multiple time scales? To this end, we used cross-sectional datasets of different ages to study the neurodevelopmental correlates of vIFC. Furthermore, the MyConnectome project in the openfMRI database contributed by Russell Poldrack supplied us with great opportunity to study the associations of vIFC with physiology and behaviors on time scales of days and weeks. In this study, we first calculated vIFC maps of 442 lifespan healthy participants from the Nathan Kline Institute/Rockland sample, as well as vIFC maps of 82 scans of 1.5-year longitudinal data from a single subject from the MyConnectome project. Then, we systematically analyzed vIFC patterns in healthy human brains across spatial and temporal dimensions. We also studied the correlations of human brain criticality vIFC with physiology and behaviors. Based on the criticality theory in nonlinear dynamics, the current study might shed new light on human brain studies from a nonlinear perspective and opens new avenues for normal and abnormal human brain studies.

## Materials and methods

### Participants and MR imaging

#### Lifespan development study

A total of 442 healthy participants were scanned using Siemens MAGNETOM TrioTim 3T scanners from two samples: (1) the Nathan Kline Institute/Rockland sample (NKI-RS, N = 126) [21–23]; and (2) the Enhanced NKI-RS sample (N = 316). Details of the MRI and participant information are summarized in Tables 1 and 2. Institutional Review Board (IRB) approval was obtained at the Nathan Kline Institute and Montclair State University. Written informed consent was obtained from all the participants or their legal guardian. All the data have been publicly shared and could be accessed via http://fcon_1000.projects.nitrc.org/indi/enhanced/data.html.

**Table 1:** MRI details of the two samples.

		NKI-Pilot	NKI-Enhanced
Scanner	Manufacturer	SIEMENS	SIEMENS
	Magnet	3T	3T
	System	TrioTim B15	TrioTim B17
M-PRAGE	TR	2500 ms	1900 ms
	TE	3.5 ms	2.52 ms
	TI	1200 ms	900 ms
	FA	8°	9°
	FoV	256 mm	250 mm
	#Slices	192	176
	Voxel Size	1×1×1 mm	1×1×1 mm
EPI	TR	2500 ms	645 ms
	TE	30 ms	30 ms
	FA	80	60
	FoV	216 mm	222 mm
	#Slices	38	40
	Voxel Size	3×3×3 mm	3×3×3 mm
	#Time Points	260	900

Abbreviations: Repetition Time [24], Echo Time (TE), Inversion Time (TI), Flip

Angle (FA), Field of View (FoV).

**Table 2:** Information of participants from the two samples.

	NKI_Pilot (N = 126)	NKI_Enhanced (N = 316)	Combined (N = 442)
Age (Years)	36.84±21.20	44.38±19.72	42.23±20.42
Age Range (Years)	7–85	8.30–83.36	7–85
Gender (Males)	68	112	180
mcBBR^1^	0.45±0.05	0.38±0.05	0.40±0.06
rmsFD^2^ [25]	0.14±0.07	0.06±0.03	0.09±0.06

^1^mcBBR is the minimal cost of the intrasubject coregistration with the boundary-based registration

^2^rmsFD is the root mean square of the frame-wise displacement for in-scanner head motion.

#### Physiological and behavioral correlates study

Ninety resting-state functional images and 14 structural images (sessions 14–104) were selected [26–28] from the publicly available openfMRI database (https://openfmri.org/dataset/ds000031/), which is an intensive assessment of a single human over a period of 18 months that includes magnetic resonance imaging and assessments of psychological function and physical health. The datasets were collected at two sites, first at the University of Texas and then at Washington University. The University of Texas determined that institutional review board (IRB) approval was not necessary. The collected datasets were transferred from the University of Texas to Washington University for analysis, and all datasets collected at Washington University were approved by the Washington University IRB. All of the images were collected on a 3T MRI scanner with a 32-channel head coil. T1-weighted data were collected using an MP-RAGE sequence (sagittal, 256 slices, 0.7-mm isotropic resolution, TE = 2.14 ms, TR = 2400 ms, TI = 1000 ms, flip angle = 8 degrees, PAT = 2, 7:40 scan time). Eyes-closed resting state fMRI was performed using a multiband echo-planar imaging (MBEPI) sequence (TR = 1.16 seconds, TE = 30 ms, flip angle = 63 degrees, voxel size = 2.4×2.4×2 mm, distance factor = 20%, 68 slices, oriented 30 degrees back from AC/PC, 96×96 matrix, 230 mm FOV, MB factor = 4, 10:00 minute scan length).

### Image preprocessing

All data preprocessing was completed with the Connectome Computation System (CCS) [29]. Detailed descriptions of the computational system can be found in our previous publications [29,30]. Preprocessing comprised both structural and functional image preprocessing. The structural image preprocessing was mainly cortical surface reconstruction [31,32] using FreeSurfer. The functional image preprocessing mainly involved the following steps: elimination of the first 4 images, correction of slice timing, alignment of each volume to a ‘base’ volume, normalization of the 4D global mean intensity into 10000, nuisance regression[33,34], bandpass (0.01–0.1 Hz) filtering, removal of both linear and quadratic trends, and alignment of the individual functional image to its anatomical image [35]. Finally, individual preprocessed 4D rfMRI time series were projected onto the fsaverage5 surface with 10,242 vertices per hemisphere and a spacing of approximately 4 mm [36]. Notably, for the single subject longitudinal data, all functional images were registered to the average surfaces of the 14 T1 images, and no slice timing correction was applied due to the use of multiband EPI.

### Quality control procedure

Quality control is a key part of solid data analysis. We considered the following steps for quality control: 1) brain extraction or skull stripping, 2) brain tissue segmentation, 3) pial and white surface reconstruction, 4) boundary-based functional image registration (BBR) to structural image, 5) head motion correction, and (6) consideration of outlier effects, and vIFCs outside two standard deviations were removed. We acquired screenshots for the first four steps and controlled their quality by visual checking. For images that brain extraction was not very good, we used two types of thresholds for the FSL BET as well as FreeSurfer automated skull strips to supply templates for further manual editing. Quantitative controls of BBR (mcBBR< = 0.5) and head motion (maxTran< = 2 mm, maxRot< = 2°, mean FD<0.2 mm) were also used.

### vIFC method

The vIFC method has been described in detail in our previous publication [5]. Considering the three properties designed in vIFC, increased temporal variations, increased within-group correlations, and decreased between-group correlations, the formula is as follows:

$$vIFC(i)=\frac{STD(i){PCC}_{in}}{{PCC}_{out}}=\frac{\sqrt{\sum_{n=1}^{N}{\left(x_{i}\left(t_{n}\right)-<x_{i}\left(t_{n}\right)>\right)}^{2}}\cdot <PCC_{ij}\left(j\in I\right)>}{<PCC_{ik}\left(k\notin I\right)>}$$ (1)

There are three types of vertices, {i}, {j ∈ I}, and {k ∉ I}. For a given vertex i, I stands for its neighborhood cluster, j represents vertices in I, and k represents vertices outside the neighborhood. x_i_ [21] is the fMRI BOLD value of vertex i at time t_n_, PCC is the intervertex Pearson correlation coefficient across time, STD is the standard deviation of the BOLD time series, and <> means averaging within the particular group. Here, we employed an assumption that spatial neighborhoods were more likely involved in the dominant group of vertices, which is similar to the definition of local functional regional homogeneity (ReHo). The calculation was repeated for each vertex, and we then obtained a vIFC map on the fsaverage5 surface for each participant. All individual vIFC maps were spatially smoothed with a Gaussian kernel with a 10 mm FWHM on *fsaverage5*.

### Neurodevelopmental correlates

We used the FreeSurfer Group Descriptor File (FSGD) for a general linear model that considered age, sex and sample as covariates with a DODS (Different Offset and Different Slope) setting. In more detail, FSGD is a way to describe a group of subjects and their demographic and behavioral data. FreeSurfer programs can automatically compute the design matrix from an FSGD file, and then mri_glmfit was used to perform the general linear model statistics. Finally, the vertex-wise significance values for each contrast of group comparisons were corrected with the FDR method (FDR **α** = 0.05/2, corrected *p* = 0.05/2).

### Physiological and anxiety correlates

Eighty-two resting state datasets with during-scan anxiety and after-scan physiological measurements, including diastolic blood pressure, systolic blood pressure, and pulse (heart rate), were used to study the physiological basis and behavioral correlates of vIFC. Anxiety during scan was measured using a seven-point survey (1 was extremely anxious and 7 was extremely good/not anxious). Similar to the above neurodevelopmental correlates study, a general linear model was constructed using the FreeSurfer FSGD file with DODS, including the day of the week, diastolic and systolic blood pressures, pulse, and anxiety as covariates. The vertex-wise significance values for each contrast of group comparisons were corrected with FDR method (FDR **α** = 0.01/2, corrected p = 0.01/2).

## Results

### vIFC in the left ventral posterior cingulate gyrus increases with age

**Fig 1:**
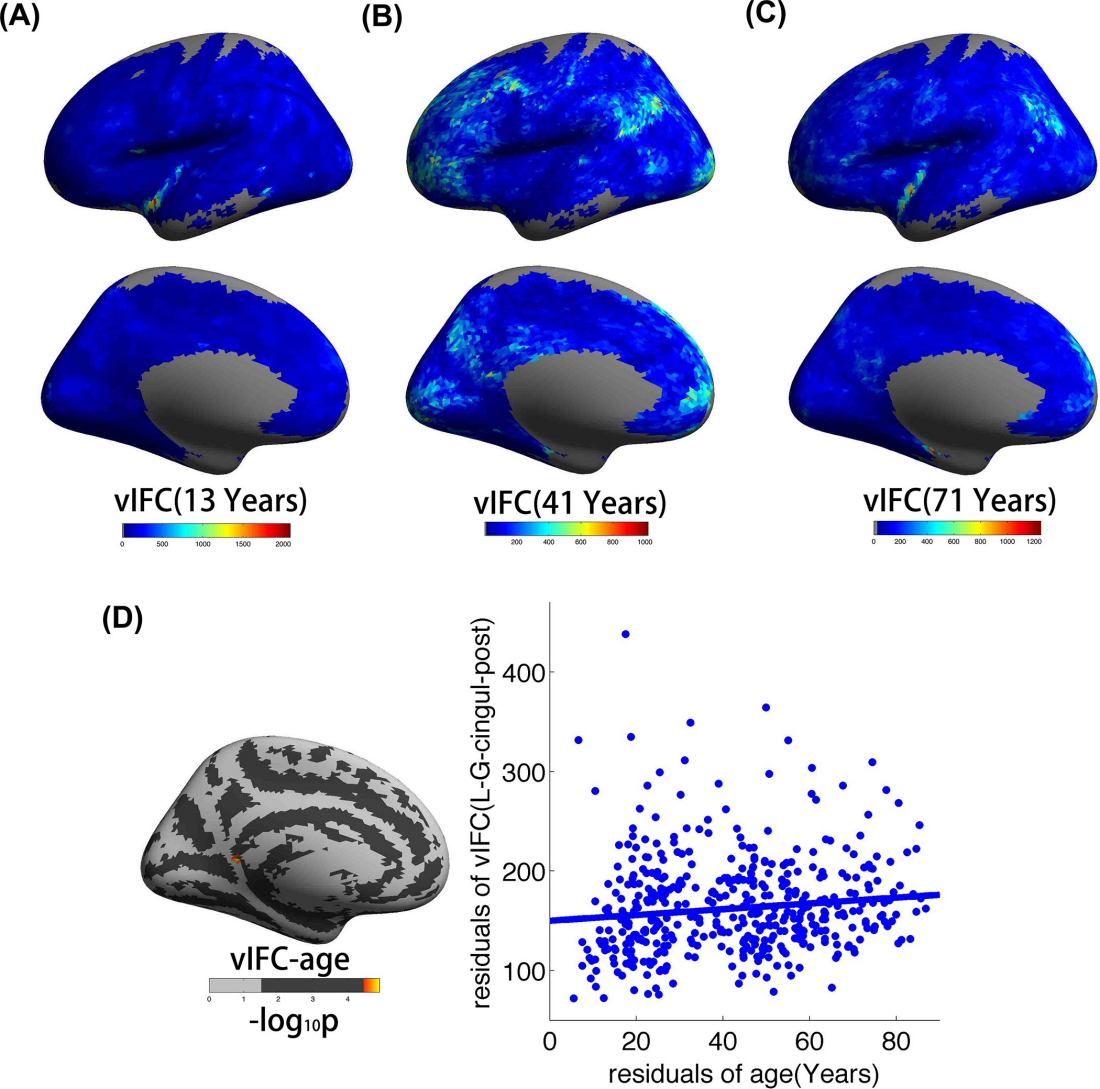
vIFC variations across the lifespan development of the human brain. (A), (B) and (C), respectively, illustrate vIFC patterns for three different subjects with ages of 13, 41, and 71 years. (D) The vertex-wise significance of age effects on vIFC using GLM statistics as well as scatterplots of the partial correlations of vIFC with age.

vIFC patterns for three subjects aged 13, 41, and 71 years are shown in Fig 1(A), 1(B) and 1(C), respectively. The older participant exhibited low vIFC values. This is consistent with our common sense that young brains contained more dynamical changes. For all three subjects, the inferior parietal lobule, precuneus, occipital pole and insula were special regions with high vIFC values. Fig 1D shows the vertex-wise significance of age effects on vIFC using GLM statistics. The vIFC of the left ventral post cingulate gyrus increased with age. For a more intuitive illustration of vIFC and age, we also plotted scatterplots of the partial correlations of vIFC with age.

### Physiological basis and anxiety correlates

**Fig 2:**
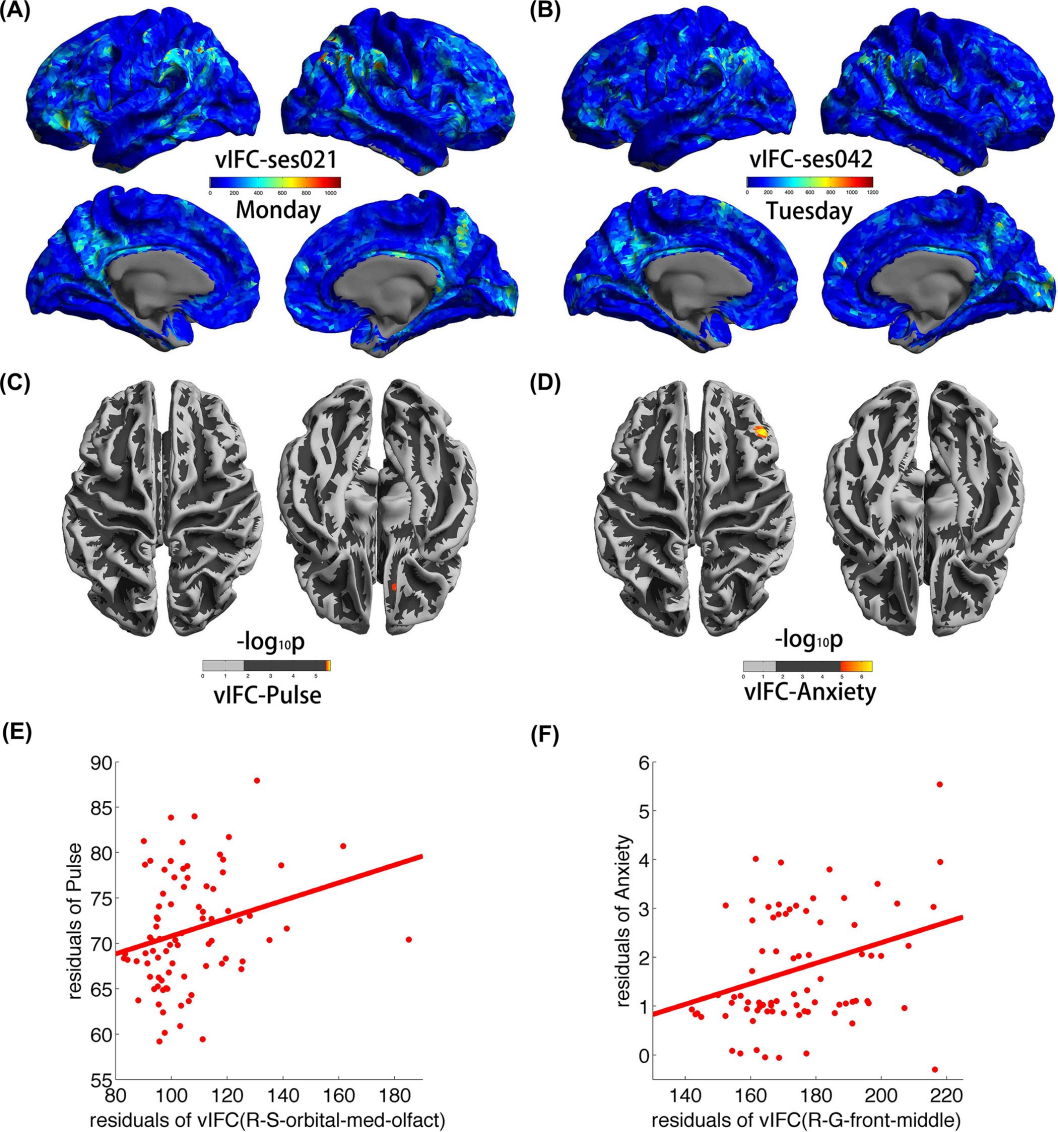
Physiological basis and anxiety correlates of vIFC. (A) and (B), respectively, illustrate vIFC patterns for sessions 21 (on Monday) and 42 (on Tuesday) of the resting state scans; (C) and (D) show significant correlations of vIFC with physiology (pulse) and anxiety. (E) Illustrated scatterplots between vIFC and pulse. (F) Illustrated scatterplots between vIFC and anxiety [37].

To give an intuitive illustration of vIFC variations across the entire cortical mantle of the single subject, Fig 2A & 2B respectively show vIFC patterns for sessions 21 (on Monday) and 42 (on Tuesday) of the resting state scans. Similar to vIFC during lifespan, the inferior parietal lobule and the precuneus exhibited high vIFC. Fig 2C & 2D show significant correlations of vIFC with physiology and anxiety, and we only found significant positive correlations represented by the warm color. In more detail, vIFC of the right S_orbital_med-olfact exhibited significant positive correlations with pulse. Similar to the significant pulse correlations, a heteromodal association region exhibited significant correlations between vIFC and anxiety, namely, vIFC of the right middle frontal gyrus exhibited significant positive correlations with anxiety. Additionally, for an intuitive illustration of the magnitudes of vIFC, pulse, and anxiety, Fig 2E and Fig 2F, respectively, show the scatterplots between vIFC and pulse as well as vIFC and anxiety. The red color represents positive correlations.

## Discussion

vIFC has successfully detected abnormalities in human brain dynamics during AD progression [5]. However, it remains unclear whether vIFC in healthy brains is associated with neurodevelopment, physiological or behavioral measurements. Using 442 lifespan cross-sectional images and a single participant longitudinal dataset, we found stable spatial patterns of vIFC across the entire cortical mantle: the inferior parietal lobule and the precuneus exhibited high vIFC. On a time scale of years, we observed that vIFC increases with age in the left ventral post cingulate gyrus. On a time scale of days and weeks, vIFC demonstrated the capacity to identify links between anxiety and the pulse of a single participant over an 18-month period (N = 82 scans). These results suggest that vIFC can serve as an efficient neuroimaging marker for detecting physiological, behavioral, and neurodevelopmental transitions. Traditional neuroimaging indexes, such as ReHo, are mostly based on linear correlations or linear statistical models. However, the realistic architecture of the human brain exhibits several spatiotemporal scales and could not be such a simple linear relationship. The current vIFC study based on nonlinear dynamics in physics may shed new light on human brain studies from a nonlinear perspective and opens new avenues for normal and abnormal human brain studies.

### Functional criticality of the human brain across lifespan development

Previous studies generally used morphological measurements, such as gray matter volume [38], cortical thickness [39], or functional measurements (i.e., ReHo and EC) [40], to construct the developmental trajectory of the human brain. However, all these measurements lack direct dynamical meanings. Thus, we used vIFC, which has a solid theoretical basis, to explore the functional criticality of the human brain across a lifespan from a dynamical and nonlinear perspective. Generally, vIFC have a decreasing trend with age. However, we did not find significant negative correlations of vIFC with age using either region-wise or vertex-wise analysis.

One of the most surprising findings was that the inferior parietal lobule and precuneus exhibited high vIFC. It is well known that the inferior parietal lobule and the precuneus are parts of the default mode network in resting state fMRI studies [25,41,42]. This result aligned with previous studies using other resting-state fMRI measurements and proved that vIFC is a sensitive index for detecting functional criticality in the human brain. vIFC in the inferior parietal lobule, more specifically, the angular gyrus, showed positive correlations with age using region-wise analysis (r = 0.1968, p = 3.23e-4<0.05/76/2). Angular gyrus has been linked to sensory integration and is vital for both social cognition and cognition [43]. Our results may underly enhancing social cognition and cognitive ability with aging. We believe that this is different from traditional aging but may indicate a more open-minded and optimistic attitude toward life itself.

We found significant positive correlations of vIFC with age in the left ventral posterior cingulate gyrus. The posterior cingulate cortex forms a central node in the default mode network of the brain and communicates with various brain networks [44]. Indeed, the posterior cingulate cortex is highly connected and one of the most metabolically active regions in the brain. Cerebral blood flow and metabolic rate in the posterior cingulate cortex are approximately 40% higher than average levels across the entire brain. Moreover, considering the posterior cingulate gyrus as a hub of brain, increased vIFC with age may reflect changes in development. This finding confirmed our algorithm and suggested vIFC as an efficient and sensitive neuroimaging marker in healthy populations.

### Physiological basis and anxiety correlates

The above results demonstrated that the vIFC method could detect a link between brain dynamics and neurodevelopment on a time scale of years. However, some disorders are characterized by onsets over a short period. Thus, we used a long-term neural and physiological phenotyping of a single subject [28] to explore the physiological basis and behavioral correlates of vIFC. Our analysis revealed that vIFC in the right S_orbital_med-olfact exhibited significant positive associations with pulse. Studies have considered pulse as a confounding factor in fMRI output [45]. There were also some studies that considered pulse as a meaningful measurement and correlated it with functional connectivity [46]. Thayer et al. even proposed heart rate variability as a marker for stress and health [37]. Our results here, based on correlations of heart rate with vIFC, elucidated the physiological basis of functional criticality in the human brain. Generally, high vIFC and moderate heart rate indicate good health. The positive correlations of vIFC with pulse in the right S_orbital_med-olfact may correspond to a subtle body workout.

Similar to the pulse correlations, a heteromodal association region exhibited significant correlations between vIFC and anxiety: Positive correlations were found in the right middle frontal gyrus. In this study, anxiety was measured with a short-term state after the fMRI scan. Previous studies consistently reported that anxiety was related to anterior cingulate cortex [47], insula [48] and prefrontal cortex [49]. Our study testified that the dynamical characteristics in the middle frontal gyrus were associated with anxiety. The middle frontal gyrus is a part of the prefrontal cortex, and the prefrontal cortex is known to be associated with executive function. Feelings of anxiety were largely related to social experiences, emotions, self-referential and collection of prior experiences, and finally these feelings of anxiety need to call on executive function to make a decision. In this condition, anxiety was involved in the emotional network and executive network, and the association between anxiety and the middle frontal gyrus was reasonable. Our study was not only consistent with a previous study [50] but also gave anxiety a dynamical underpinning of the human brain. In summary, our study confirmed vIFC as an efficient biomarker for human brain studies and emphasized the importance of human brain criticality in shaping cognitive abilities.

## Conclusions

In this work, we applied the vIFC method to explore the associations of human brain criticality with physiology, behavior, and neurodevelopment. We found consistent vIFC patterns in healthy human brains across a lifespan: high vIFC in the inferior parietal and precuneus. Patterns characterized by vIFC were largely similar to patterns identified by other resting state fMRI metrics. We also confirmed the associations of vIFC with base heart rate, anxiety and age. Combining nonlinear dynamics and fMRI methodologies, our vIFC study sheds new light on human brain studies from the perspective of nonlinearity and indicates the neuropsychological and neurophysiological meanings of vIFC.
